# The Immune Landscape of Hepatitis B Virus-Related Acute Liver Failure by Integration Analysis

**DOI:** 10.1155/2022/6764379

**Published:** 2022-01-06

**Authors:** Jiao Gong, Yaqiong Chen, Jing Cao, Yang Wang, Jiahao Chen, Danyang Li, Liuping Sha, Xinhua Li, Yutian Chong, Bo Hu

**Affiliations:** ^1^Department of Laboratory Medicine, Third Affiliated Hospital of Sun Yat-sen University, Guangzhou, China; ^2^Department of Infectious Diseases, Key Laboratory of Liver Disease of Guangdong Province, Third Affiliated Hospital of Sun Yat-sen University, Guangzhou, China

## Abstract

Hepatitis B virus-related acute liver failure (HBV-ALF) is a common type of liver failure, associated with high short-term mortality and morbidity rates. However, the immune landscape of HBV-ALF and its correlation with cell death are currently unknown. Based on 3 Gene Expression Omnibus data sets, infiltrated immune cells were quantified by single-sample gene set enrichment analysis method. The expression levels of immune genes and the abundance of immune cells in liver failure were compared with those in normal liver. The enrichment scores of cell death gene sets from Kyoto Encyclopedia of Genes and Genomes (KEGG) were calculated by gene set variation analysis method, and a protein-protein interaction (PPI) network was constructed using Cytoscape. Besides 21 differentially expressed immune genes, we identified 11 types of differentially infiltrated immune cells in HBV-ALF compared with normal liver. Enriched pathways of these immune genes mainly consisted of chemokine receptors, chemokine binding, interleukin-10 signaling, and TNFs bind their physiological receptors by Reactome pathway analysis. In addition, the enrichment scores of apoptosis and necroptosis pathway instead of autophagy and ferroptosis were increased in liver failure compared with normal liver. PPI network and gene cluster analysis of immune genes and apoptosis and necroptosis genes suggested that hub genes were mainly related to immune response and apoptosis. In summary, our study offers a conceptual framework to understand the immune landscape of HBV-ALF, which might help to improve prognosis.

## 1. Introduction

Acute liver failure (ALF), a life-threatening illness, is referred as a clinical syndrome with acute and massive hepatocellular destruction within 4 weeks. The features of ALF include acute development of jaundice, multiple organ failure, and/or encephalopathy in patients without a history of liver disease [1, 2]. The short-term mortality of ALF can reach up to 40-50%, and the main death causes in patients with ALF are largely ascribed to sepsis with multiple organ failure and cerebral herniation due to increased intracranial pressure [1, 3].

Hepatitis B virus (HBV) is the main cause of ALF in Eastern countries [4]. According to World Health Organization (WHO) report, 257 million people were infected with chronic hepatitis B in 2015 [5]. Reportedly, more than 0.1%-0.5% patients with HBV infection would develop ALF [6, 7]. However, current available treatments for HBV-related acute liver failure (HBV-ALF) are very limited and inconvenient, such as antiviral therapy and artificial liver treatment; liver transplantation is effective but expensive, and liver grafts are very limited [7, 8]. A comprehensive understanding of the etiology of HBV-ALF, improvement of treatments, and intensive care support will shed more light on dramatically improving the prognosis of HBV-ALF patients.

Although the therapeutic potential of cell death in cancer has attracted increasing attention, the change of apoptosis, autophagy, ferroptosis, and necroptosis pathways are still unknown. Cell death is at the center of virtually every acute and chronic liver disease [9]. Necroptosis depends on kinase activities of receptor interacting proteins (RIPs) with necrotic morphology, regulated by RIPK1, RIPK3, and mixed lineage kinase domain-like protein (MLKL) [10]. Ferroptosis, a novel type of necrosis, is characterized by the accumulation of intracellular iron ions, rise of lipid peroxidation, mitochondria, and mitochondrial membrane density [11]. Elevated levels of oxidative stress in ALF could promote disease progression [12]. Therefore, simultaneously inhibiting multiple cell death pathways may be required to alleviate ALF.

So far, the pathological mechanisms of HBV-ALF are not well illustrated. Previous ALF-related studies mainly focused on the function of a single gene or a cell type, viral itself or interactions between viral and host [6, 7, 13–19]. For instance, during the development of ALF, upregulation of IL-1*α*, IL-1*β*, and IL-18 activated NF-*κ*B pathway by downregulating inhibitor of kappa B levels, promoted the secretion of IL-6 and TNF*α*, which resulted in apoptosis, and ultimately to massive hepatocellular destruction [13–15]. Huadi et al. found a fierce immune response in HBV-ALF. However, they obtained the differentially expressed genes (DEGs) by intersecting DEGs from individual gene sets. In this research, based on 3 datasets, we integrated the raw data and comprehensively displayed the immune landscape of HBV-ALF and identified the upregulated activities of apoptosis and necroptosis pathway in liver failure compared with normal liver. PPI network analysis revealed the enrichment of immune response and apoptosis.

## 2. Materials and Methods

### 2.1. Dataset Collection

All gene expression profiling data with GPL570 platform (GSE14668, GSE38941, and GSE96851) [20–22] were obtained from Gene Expression Omnibus (GEO, http://www.ncbi.nlm.nih.gov/geo/) database. We got 89 samples, found 45 duplicates, and finally got 44 samples (control = 27, LF = 17). Besides, all the 17 LF samples were from 4 patients. Healthy liver samples were from HBV-negative liver donors. After removing duplicated data, we integrate raw data performed the quality control and adjustment using simpleaffy and affyPLM R package. Moreover, we performed Principal Component Analysis (PCA) analysis on these datasets. The optimal number of clusters (K) was generated according to “wss” (for total within sum of square) and visualized by “factoextra” package of R. We first conducted PCA analysis on all the dataset and found that they can be divided into two categories, liver failure and control group (Supplementary figure 1 A and B).

Immune genes encoding immunomodulators and chemokines were downloaded from TISIDB—an integrated repository portal for tumor-immune system interactions [23]; gene signatures for calculating immune cell types were from Charoentong et al. research [24]. Metagenes are characterized by nonoverlapping sets of genes that are representative for specific immune cell subpopulations [24]. Moreover, metagenes are neither expressed in tumor cell lines nor in normal tissue [24]. The expression of these metagenes was then used during gene set enrichment analysis [24]. Thus, these calculations were less sensitive to noise resulting from sample impurity or sample preparation compared with deconvolution methods.

### 2.2. Functional Enrichment Analysis and Protein-Protein Interaction Network Analysis

As previously described, the gene ontology analysis and Reactome pathway analysis were done by R package “clusterProfiler,” and a *p* value < 0.05 was significant [25, 26]. The differentially expressed immune genes were loaded to STRING database to construct the PPI diagram, and then, cytoscape plug-in, Molecular Complex Detection (MCODE), and CytoHubba were utilized to develop PPI network. The default settings of MCODE were degree = 2, node score = 0.2, *K* core = 2, and a maximum depth of 100. The value of “degree” was used to estimate hub genes by CytoHubba.

### 2.3. Statistical Analysis

The difference between HBV-ALF and normal control was analyzed using *t*-test. All statistical analyses were performed by R (version 4.0.1). Heatmaps were constructed using Complexheatmap R package, and the correlation plot was developed using corrplot R package.

## 3. Results

### 3.1. Immune Interface of Immune-Related Molecular and Cells in Liver Failure

**Figure 1:**
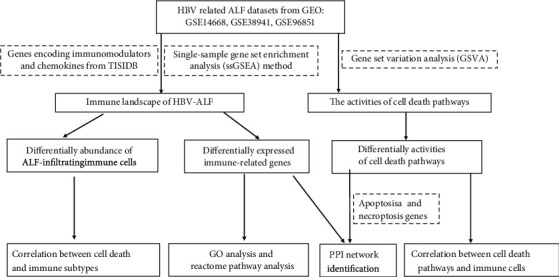
Flowchart of our research.

**Figure 2:**
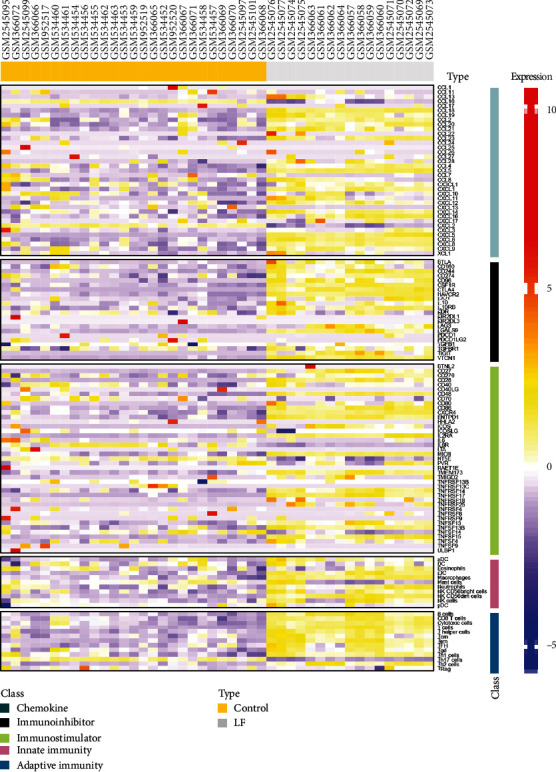
Heatmap of immune-related genes and cells in HBV-ALF and normal control. The integration analysis of GSE14668, GSE38941, and GSE96851. The expression of immune genes and infiltrated immune cell composition was analyzed. With single-sample gene set enrichment analysis (ssGSEA) method, we obtained 24 types of infiltrated immune cells. LF: HBV-ALF; control: normal control.

To comprehensively analyze the immune landscape in HBV-ALF, we first obtained immune genes encoding immunomodulators and chemokines from TISIDB, gene signatures for calculating immune cell types from previous research [24], and 3 gene expression profiling data (GSE14668, GSE38941, and GSE96851) from GEO database. The flowchart of our study is depicted in Figure 1. Using single-sample gene set enrichment analysis (ssGSEA) method and integration of these GEO arrays, we obtained 24 types of infiltrated immune cells, which included innate immune cell types and adaptive immune cell types. Then, we further mined the difference in those abovementioned immune genes and cells between normal liver tissues and samples from HBV-ALF patients. As shown in Figure 2, genes encoding chemokine, immunoinhibitor, immunostimulator, and cells from innate immunity and adaptive immunity were frequently upregulated in liver tissues of patients with HBV-ALF.

**Figure 3:**
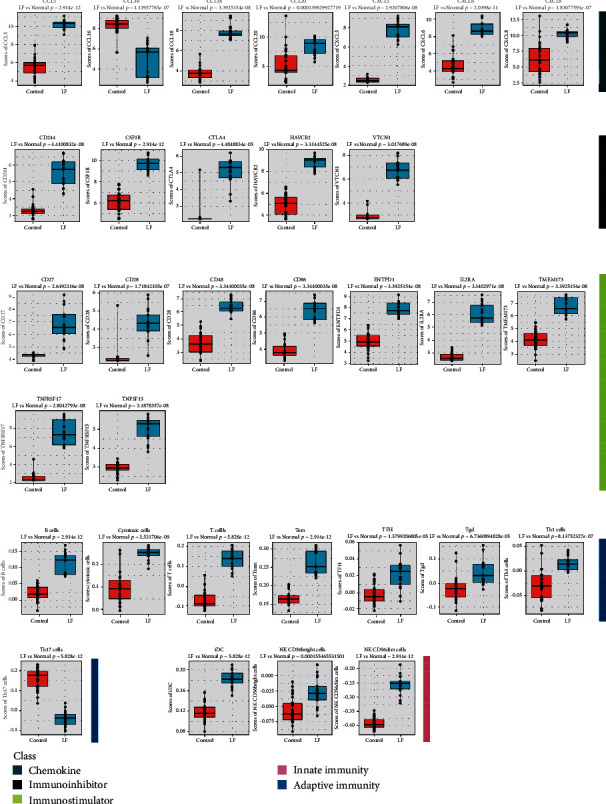
The differentially expressed immune genes and infiltrated immune cells. Comparison of immune genes and immune cells between liver tissues with HBV-ALF and normal liver.

**Figure 4:**
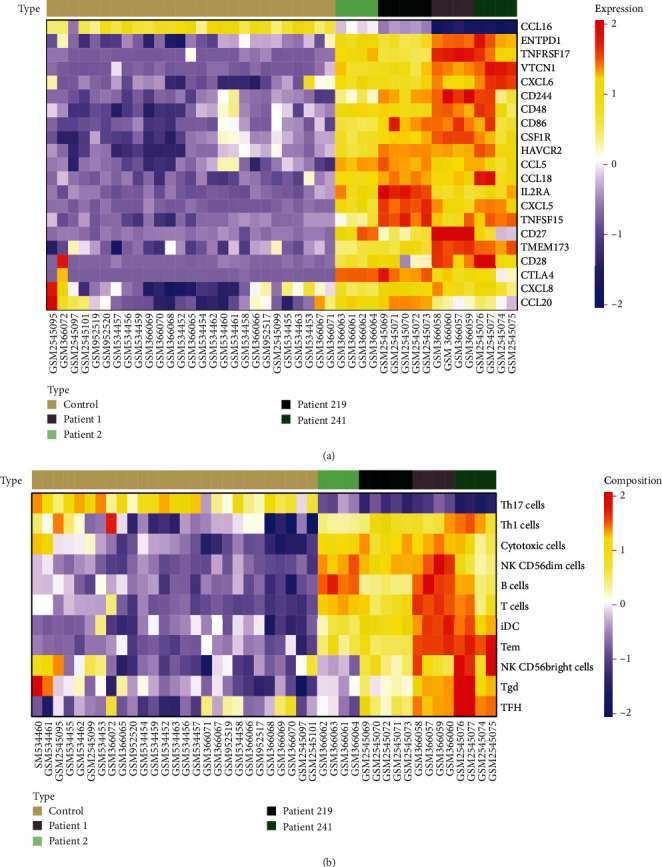
Immune status of HBV-ALF. Different patients in HBV-ALF presented with differentially expressed genes and infiltrated immune cell composition. Heatmaps of immune-related genes (a) and immune cells were depicted in HBV-ALF.

Next, we screened for the most significantly differentially expressed immune genes and infiltrated immune cell with parameters such as fold change cutoff of 1.5 and *p* value cutoff of 0.05 for *t*-test (Figure 3). The expression levels of chemokines were upregulated in HBV-ALF tissues, such as CCL5, CCL16, CCL18, CCL20, CXCL5, CXCL6, and CXCL8, while immunoinhibitors levels of CD244, CSF1R, CTLA4, HAVCR2, and VTCN1, immunostimulators such as CD27, CD28, CD48, CD86, ENTPD1, IL2RA, TMEM173, TNFRSF17, and TNFSF15. Furthermore, the number of infiltrated adaptive immune cell types and innate immune cell type, for instance, Tgd, B cells, T cells, and iDC, greatly increased. Furthermore, we conducted PCA analysis base on these genes and cells and found that they can divide cases into two categories, liver failure and control group (Supplementary figure 2 A and B). Based on these genes and immune cells, we observed that even in HBV-ALF, different patients presented with differentially expressed genes and infiltrated immune cell composition (Figures 4(a) and 4(b)). Among the patients, somebody had the highest expression of immunostimulators (TNFRSF17, ENTPD1, CD28, CD48, CD86), TFH, Tgd, Tem, iDC, cytotoxic cells, T cells, and B cells, implying an “immune-hot” status, while somebody had the lowest expression of immunostimulators but relative high expression of chemokines (CCL5, CCL20), indicating an “immune-cold” status.

### 3.2. Function and Pathway Enrichment Analyses of Differentially Expressed Immune-Related Genes

**Figure 5:**
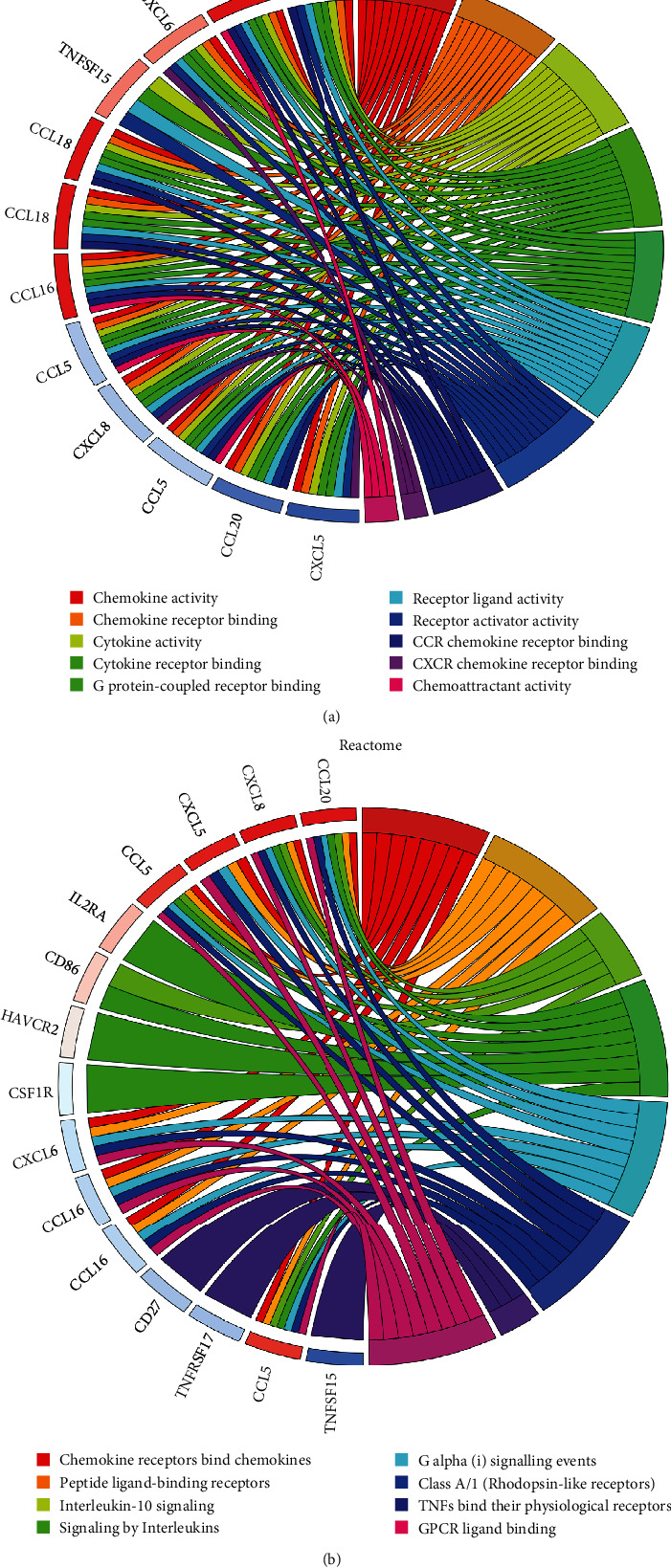
Molecular Function (MF) enrichment and Reactome pathway analysis. Molecular Function (MF) enrichment analyses (a) and Reactome pathway analysis (b) of differentially expressed immune genes.

To dig deep into the biological functions of the differentially expressed immune-related genes, we performed Molecular Function (MF) enrichment analyses of gene ontology term enrichment and Reactome pathway analysis (Figure 5). MF term was mostly linked to chemokine activity, chemokine receptor binding, cytokine activity, cytokine receptor binding, and G protein-coupled receptor binding (Figure 5(a)). Reactome pathway analysis showed enriched pathways of these genes mainly consisted of chemokine receptors bind chemokines, interleukin-10 signaling, signaling by interleukins, and TNFs bind their physiological receptors (Figure 5(b)).

### 3.3. Correlation between Cell Death and Immune in Liver Failure

**Figure 6:**
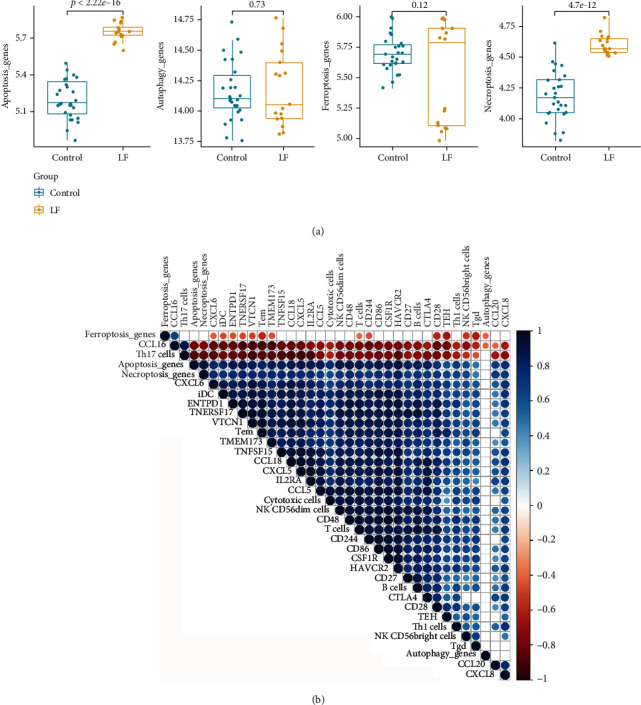
The correlation between immune and cell death. According to the apoptosis, autophagy, ferroptosis, and necroptosis gene sets from KEGG (hsa04210, hsa04140, hsa04217, hsa04216), we estimated the enrichment score (ES) of these pathways by gene set variation analysis (GSEA). (b) The correlation between immune genes, cells, and enrichment score of cell death. *p* > 0.05 was blanked from the plot.

Although the therapeutic potential of cell death in cancer has attracted increasing attention [27], the change of apoptosis, autophagy, ferroptosis, and necroptosis pathway is still unknown. Hence, we further systematically analyzed its correlation with HBV-ALF. Based on apoptosis, autophagy, ferroptosis, and necroptosis gene sets from KEGG (hsa04210, hsa04140, hsa04217, hsa04216), we estimated enrichment score (ES) of these gene sets by gene set variation analysis (GSVA) (Figure 6(a)). The activities of apoptosis and necroptosis pathway were increased in liver failure compared with normal liver. Consistent with these results, enrichment score of apoptosis and necroptosis pathway correlated with differentially expressed immune genes and immune cells (Figure 6(b)), such as CXCL6, CD28, and T cells.

**Figure 7:**
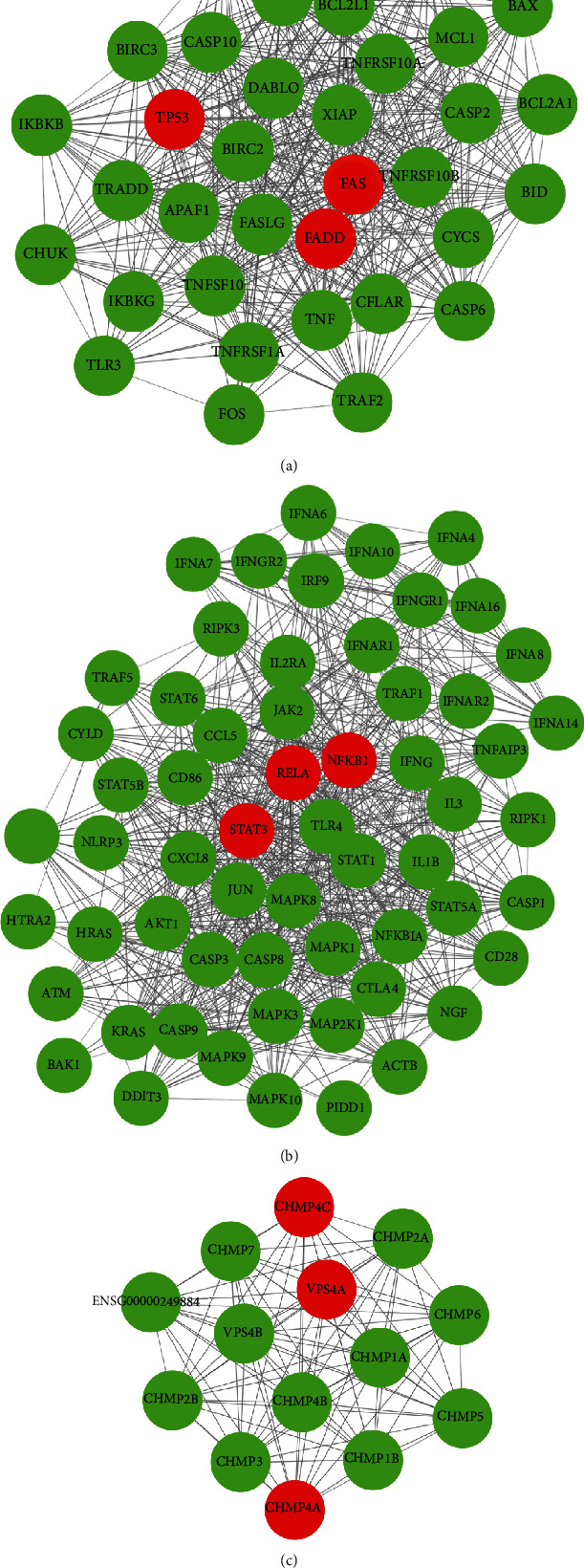
The protein-protein interaction network of immune genes and cell death genes. (a–c) Protein-protein interaction network (PPI) was constructed and imported into Cytoscape for further analysis of the MCODE plug-in, and the top 3 clusters were selected, and its hub genes in each cluster were calculated by values of degree.

To identify the core genes from apoptosis and necroptosis gene sets and the differentially expressed immune genes in the HBV-ALF tissues, we performed a protein-protein interaction network (PPI) construction and imported its results into Cytoscape for further module analysis by the MCODE plug-in. The top 3 clusters were selected, and its hub genes in each cluster were calculated by values of degree (Figure 7). The top 3 hub genes from cluster 1 were FAS, FADD, and TP53, mainly related to apoptosis (Figure 7(a)); cluster 2 was STAT3, RELA, and NFKB1 closely related to inflammation and apoptosis (Figure 7(b)); cluster 3 was CHMP4C, VPS4A, and CHMP4A, mainly linked to biosynthesis of endosomes (Figure 7(c)).

## 4. Discussion

The immune system plays a crucial role in the fate of HBV-ALF patients. In this study, we utilized bioinformatical strategies to offer a thorough view of immune-related molecular and cells, to significantly improve understanding of the immune landscape of HBV-ALF and refine current treatment strategies. The activities of apoptosis and necroptosis pathway were increased in liver failure compared with normal liver. PPI network analysis revealed the enrichment of immune response and apoptosis. Therefore, these pathways might play an important role in the pathogenesis of HBV-ALF.

Previous ALF-related studies mainly focused on the function of a single gene or a cell type, viral itself or interactions between viral and host, while our study comprehensively evaluated overall immune level in HBV-ALF. Reportedly, during development of ALF, upregulation of IL-1*α*, IL-1*β*, and IL-18 activated NF-*κ*B pathway by downregulating inhibitor of kappa B level, promoted secretion of IL-6 and TNF*α*, and then thus resulted in apoptosis and ultimately to massive hepatocellular destruction [13–15]; TNF-*α*/HMGB1 inflammation signaling pathway played an important role in pyroptosis during ALF [16]. In addition, macrophages and NK cells distributed more in HBV-ALF liver tissues compared with normal liver [7]. A number of viral factors have been identified to be associated with the progression of ALF, for example, increased variability in HBsAg preS2 region and HBV core, and host factors, such as the presence of certain human leukocyte antigen (HLA) class II locus alleles also related HBV-ALF progression [6, 17–19]. Unlike the above studies, we identified differentially expressed genes (7 chemokines, 5 immunoinhibitors, 9 immunostimulators) and the distinct abundance of immune cells in HBV-ALF compared with normal control.

Simultaneous activation of both pro- and anti-inflammatory mediators plays an important role in the early response to liver injury in ALF; the balance of these mediators determines the degree of liver injury. Following peak hepatotoxicity, the cytokine milieu is altered in order to favor the resolution of acute inflammation and promote regeneration. Interestingly, in our study, the expression of immunoinhibitors and immunostimulators was simultaneously increased in HBV-ALF. We speculated that in order to counteract the overactivated immune response, the body initiated a negative feedback by increasing the expression of immunosuppressive molecules, thereby reducing the immune-induced death of hepatocytes. Cell death is at the center of virtually every acute and chronic liver disease [9]. Our study implies that the balance of immune in liver failure should be taken into account in further study and clinical practice. The discovery of novel modes of cell death such as ferroptosis and necroptosis explains the lack of widespread use of caspase inhibitors in clinical hepatology to some extent [9]. Another intriguing result was the increased activities of apoptosis and necroptosis pathway in liver failure instead of autophagy and ferroptosis, implying the important role of necroptosis.

We also observed that even in HBV-ALF, different patients presented with differentially expressed genes and infiltrated immune cell composition. Somebody had the highest expression of immunostimulators (TNFRSF17, ENTPD1, CD28, CD48, CD86), TFH, Tgd, Tem, iDC, cytotoxic cells, T cells, and B cells, implying an “immune-hot” status, while somebody had the lowest expression of immunostimulators but relative high expression of chemokines (CCL5, CCL20), indicating an “immune-cold” status. Each status might prelude to a distinct outcome, and hence, the need for different treatment strategies depends on the status present.

In a study by Farci et al. [20], liver tissue was collected at the time of liver transplantation in two patients with HBV-associated ALF, characterized by an overwhelming B cell response apparently centered in the liver. Similarly, in our study, we also found significant difference B cell scores between the LF group and the control group. However, in the study by Farci et al., gene expression analysis was performed on data from only 2 cases while our bioinformatics analysis was performed on a relatively greater sample size. Moreover, in our study, we found 8 types of differentially expressed adaptive immune cells, and hub genes were found to be associated with the immune system and apoptosis.

Our study had some shortcomings. First, the liver samples with HBV-ALF were very hard to collect. Therefore, sample size was not large. Second, we did not verify the differential expression of genes and composition of immune genes. And we did not compare the data with the simple HBV group; some of the immune changes from HBV infection may affect the result. Also, we did not utilize other methods to estimate immune cell. Hence, more studies with large sample size will be needed to further validate our findings.

In summary, we identified the differentially expressed immune genes and infiltrated immune cells in liver samples of HBV-ALF compared with normal liver and discovered the increased activities of apoptosis and necroptosis pathway in liver failure. PPI network analysis revealed the enrichment of immune response and apoptosis. Our findings highlight that greater emphasis should be placed on the immune landscape of HBV-ALF to improve prognosis.

## Data Availability

The data used to support the findings of this study are included within the article.

## Abbreviations

ALF: Acute liver failure
HBV: Hepatitis B virus
ssGSEA: Single-sample gene set enrichment analysis
KEGG: Kyoto Encyclopedia of Genes and Genomes
PPI network: Protein-protein interaction network.

## Supplementary Materials

Supplementary Materials Supplementary Figure 1: Principal Component Analysis (PCA) analysis of genes. (A) The optimal number of clusters (*K*) was selected with factoextra package. (B) Visualization of cluster results using factoextra. Supplementary Figure 2: Principal Component Analysis (PCA) analysis of the most significantly differentially expressed immune genes. (A) The optimal number of clusters (*K*) was selected with factoextra package. (B) Visualization of cluster results using factoextra.
