# Supplementary material for: The Immune Landscape of Hepatitis B Virus-Related Acute Liver Failure by Integration Analysis
Source: J Immunol Res. 2022 Jan 6;2022:6764379. doi: 10.1155/2022/6764379 (PMC8758293; doi:10.1155/2022/6764379)
Supplement: Supplementary Materials — Supplementary Figure 1: Principal Component Analysis (PCA) analysis of genes. (A) The optimal number of clusters (K) was selected with factoextra package. (B) Visualization of cluster results using factoextra. Supplementary Figure 2: Principal Component Analysis (PCA) analysis of the most significantly differentially expressed immune genes. (A) The optimal number of clusters (K) was selected with factoextra package. (B) Visualization of cluster results using factoextra. [file 6764379.f1.docx]

**SFigure 1**


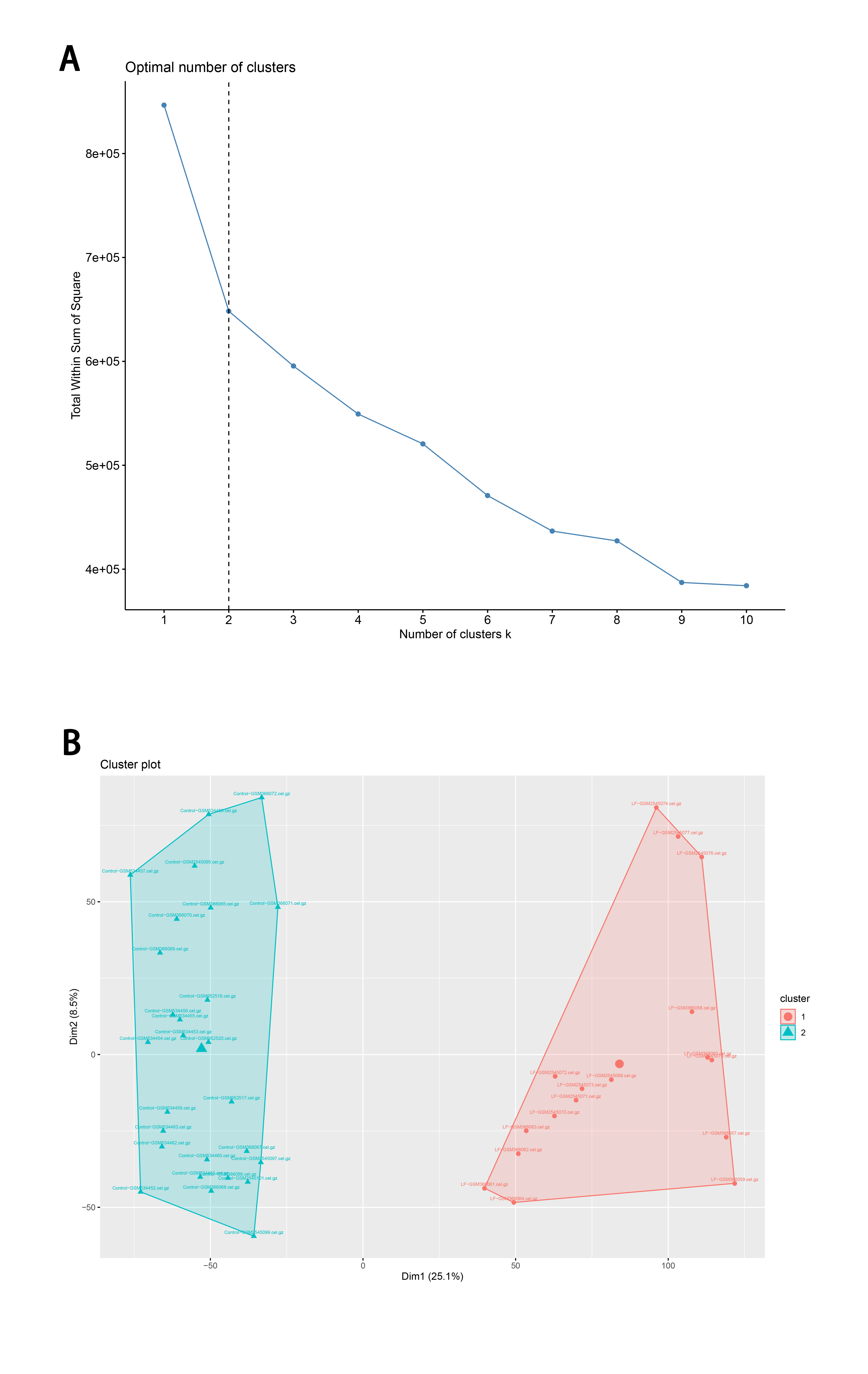


**Supplementary figure 1.** Principal Component Analysis (PCA) analysis of genes. (A) The optimal number of clusters (K) was selected with factoextra package. (B) Visualization of cluster results using factoextra.

**SFigure 2**


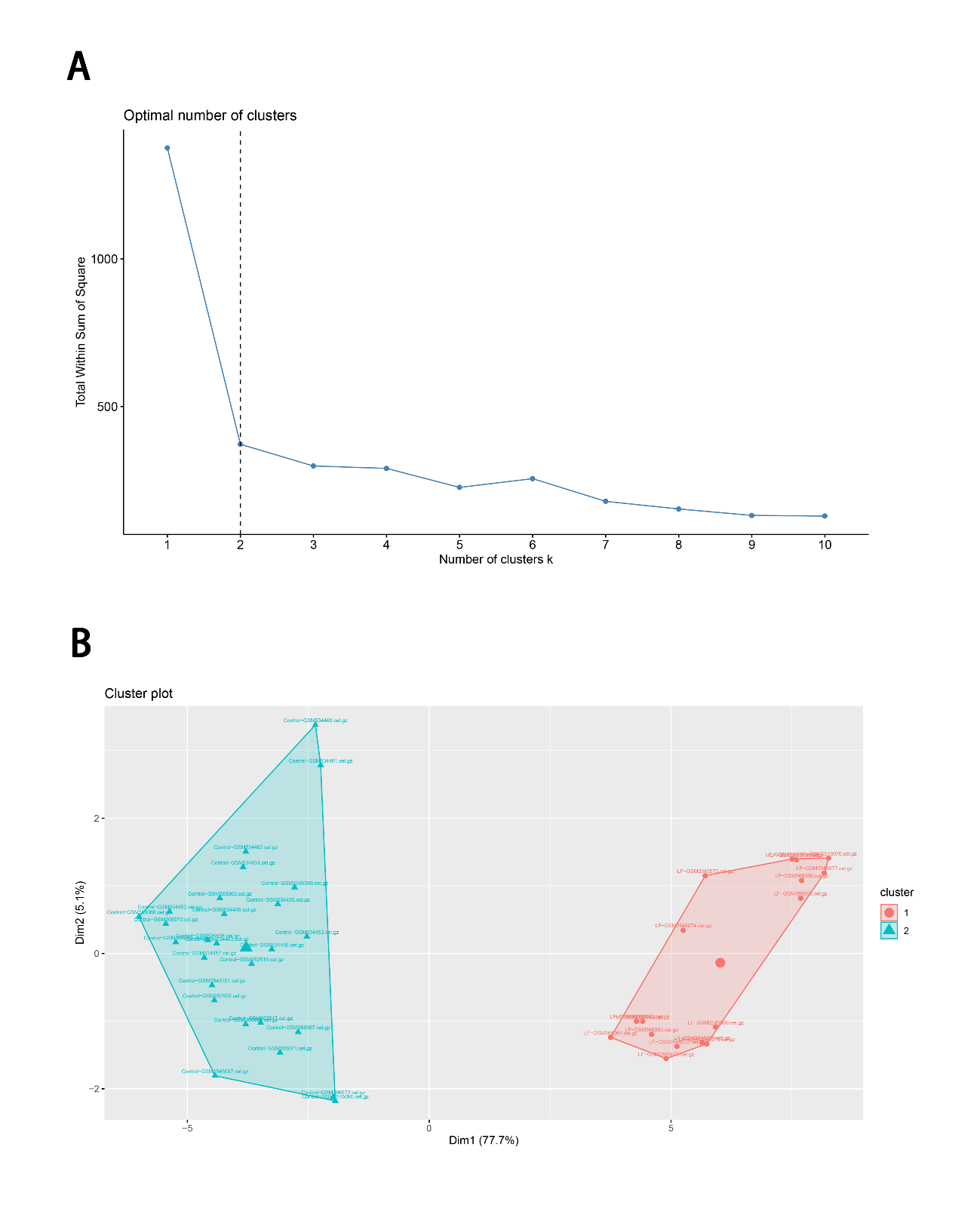


**Supplementary figure 2.** Principal Component Analysis (PCA) analysis of the most significantly differentially expressed immune genes. (A) The optimal number of clusters (K) was selected with factoextra package. (B) Visualization of cluster results using factoextra.
